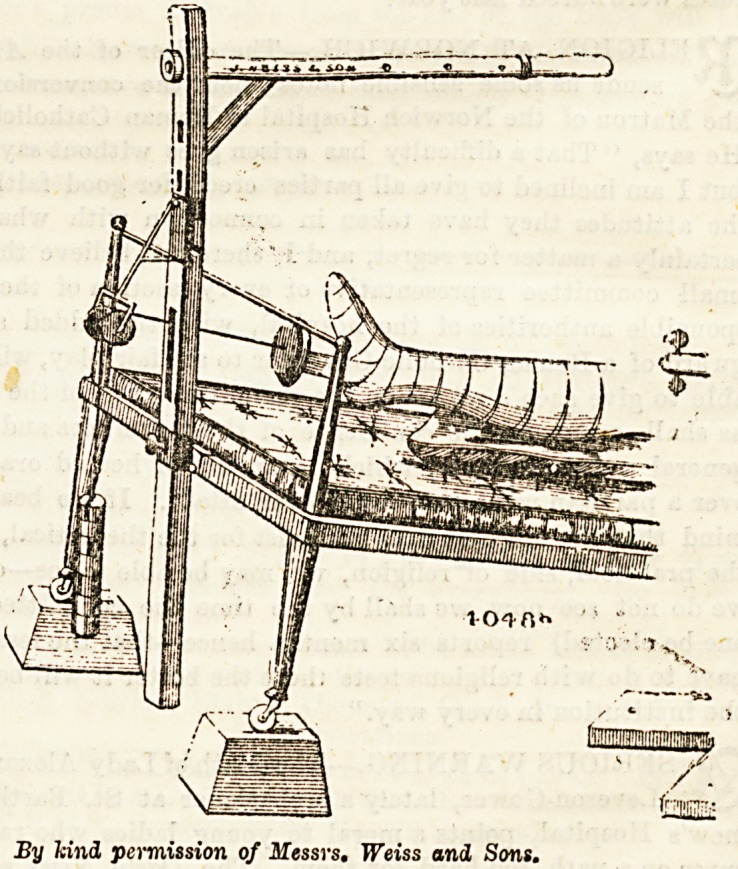# The Hospital Nursing Supplement

**Published:** 1891-04-25

**Authors:** 


### "The Hospital" Nursing Mirror.

Being the Extra Nursing Supplement of "The Hospital" Newspaper.

Contributions for this Supplement should be addressed to the Editor, The Hospital, 140, Strand, London, W.C., and should have the word "Nursing" plainly written in left-hand top corner of the envelope.

### En Passant.

INFLUENZA AGAIN.—There can be no doubt that the Russian influenza is epidemic again in the Midlands; nurses at Sheffield are already over-worked in consequence. We would warn all nurses to pay particular attention to their health, that they may not be laid up just when their services are most needed.

WORK IN WALES.—The first annual report of the Welsh Branch of the Queen Victoria Institute is interesting. Full particulars of the rise of the association and the work it is doing are given; there is also a page of specimen cases. Rules for the engagement of nurses, and then a sketch of the advantages and methods of district nursing, fill up the pamphlet. The Branch has its head-quarters at Cardiff, Miss J. E. Heath is Lady Superintendent and Dr. Alfred Sheen is Secretary and Treasurer. Over 200 cases were nursed last year.

RELIGION AT NORWICH.—The editor of the *Argus* sends us some sensible notes anent the conversion of the Matron of the Norwich Hospital to Roman Catholicism. He says, "That a difficulty has arisen goes without saying, but I am inclined to give all parties credit for good faith in the attitudes they have taken in connection with what is certainly a matter for regret, and I, therefore, believe that a small committee representative of every section of the responsible authorities of the hospital, with the added safeguard of a Roman Catholic Governor to see fair play, will be able to give such a report on the various bearings of the case as shall command the confidence of the Governors and the general public in a way which no amount of heated oratory over a partisan resolution could ever attain. If we bear in mind that the hospital does not exist for the theoretical, but the practical, side of religion, we may be able to see—or if we do not see now, we shall by the time the committee (if one be elected) reports six months hence—that the less we have to do with religious tests there the better it will be for the institution in every way."

A SERIOUS WARNING.—The death of Lady Alexandra Leveson-Gower, lately a probationer at St. Bartholomew's Hospital, points a moral to young ladies who rashly enter on a path too hard for them. The *Daily News* says: "Gently nurtured and shielded from sight or hearing of the deeper sorrows of the world and the darker phases of its sins, these young creatures ardently rush forth to the work of nursing and the daily toil that is a necessary part of the profession. When health begins to suffer and energies to fail the spirit is still willing, and a reluctance to confess themselves vanquished induces them to persevere until all power of recuperation is lost. It was so with Lady Alexandra. Her grief on the death of her mother, the late Duchess of Sutherland, which occurred in November, 1888, turned her thoughts to active work in the world, and she soon after became nurse in a London hospital, scrubbing and drudging as it is considered necessary that nurses should. Her health soon began to give way under the hard work, food so different from that to which she had been accustomed in her luxurious home, and the depression that is inevitable upon the contact with so much suffering. For many months Lady Alexandra had been lying ill at Argyll Lodge, the residence of her uncle, the Duke of Argyll."

ANOTHER NURSING CHART.—William Andrews, of Hull, has issued another copyright nursing chart, for recording all the treatment and symptoms of a patient for twenty-four hours. The chart is very neat and nicely printed, but we can see no very distinguishing features about it. The price is 5s. a hundred, or 25 for 1s. 6d.

SHORT ITEMS.—Stirling District Nursing Association has decided to affiliate with the Queen Victoria Institute.—Stamford Nursing Society nursed 150 patients last year. There is work for two permanent nurses, but the income was only £80.—There will be no Local Government Board inquiry into the working of the Fulwood Infirmary. The report of the sub-committee is deemed sufficient.—A lively correspondence is going on in the Norfolk papers as to whether the Matron of the hospital ought or ought not to resign now she has become a Roman Catholic.—The Empress of Germany was present at the consecration of Sister Auguste Hertzer, who nursed Emin Pacha through his late illness.—Worcester City and County Institution has started a maternity branch at Wychside, Malvern.—The Queen of Sweden has given 300 kr. to the Red Cross Home for Sick Nurses at Stockholm.

PRESENTATIONS AT BURNLEY.—Four of the nurses of the Victoria Hospital, Burnley, lately won their certificates, and the occasion was marked by considerable ceremony. Tea was served in the nurses' room, and then Dr. Brown delivered an address explaining that two of the doctors and the Matron had given lectures and otherwise trained the nurses till several of them had attained efficiency. He could speak in the highest terms of the Matron, Miss Piggott, and her method of training. Mr. Ecroyd then gave the prizes and certificates to the recipients as follows: Nurse D. C. Ville, certificate and two prizes, consisting of a choice copy of Shakespeare's works, in case, and a copy of "Haydn's Dictionary of Medical Knowledge"; Nurse Grant, certificate and prize, consisting of a copy of Tennyson's works bound in morroco; Nurse Bowen, certificate; and Nurse Foster, certificate. Some of the ladies present also presented the nurses with bouquets, and the whole ceremony was very pretty and cheerful.

NORWICH HOSPITAL.—The following scheme for securing fairer remuneration to the nurses has been adopted at the late meeting of the supporters of the Norfolk and Norwich Hospital:—

1. That all nurses of the Hospital who have received three years' training and are willing to bind themselves for a further period of three years, and also the head nurses after three years' service, shall be entitled to a percentage on their salaries, by way of bonus, placed to their credit at the end of each year, at the following rates: For those engaged in private nursing only, at 10 per cent.; for those engaged partly in private and partly in hospital nursing, at 7½ per cent.; for those engaged in hospital nursing only, at 5 per cent.

2. These sums to be placed to the credit of the nurse in the Nurses' Fund, now called the Superannuation Fund, but in future to be called the Norfolk and Norwich Hospital Nurses' Fund, and to bear interest at 5 per cent. as in the case of her own savings.

3. In the event of a nurse joining the National Pension Fund the amount credited to her may, at her request, be transferred to augment the premium paid by her.

4. Should any nurse, to whom any bonus has been credited, break her engagement, or be dismissed from the Hospital, she shall forfeit all claim to receive such bonus, the amount of which shall revert to the Hospital funds, unless for any special reason the Board shall otherwise determine.

### Lectures on Surgical Ward Work and Nursing.

By Alexander Miles, M.B. (Edin.), C.M., F.R.C.S.E.

### LECTURE XXI.—REST.

In this lecture I begin the description of various appliances and apparatus which are used in surgery, in very different conditions, and under varying circumstances, but all with the same primary end in view, namely, the securing and maintaining of rest. By this term in surgery is meant something more than the word indicates in ordinary language. Periodic rest is a physiological condition, and it is during these periods that we recuperate our worn-out bodies and brains, and so prevent them passing from a state of health to one of disease. For example, after a hard day's work in your wards you may find your limbs ache, your feet sore and swollen, or even blistered, and as an evidence of brain fatigue you have headache, but you know that a few hours' rest in the sitting-room, some light reading, and then your night's sleep will remove all these unpleasant symptoms. If rest be such an efficient means of treating these simple conditions, it is certainly no less valuable when you have to deal with conditions which are more serious. John Hunter long ago said that "the first and great requisite for the restoration of injured parts is rest," and surgeons have all come to acknowledge the wisdom of this dictum, and to act on it. It is by rest that such common affections as strumous disease of joints, fractures of limbs, disease of the spinal column are mainly treated. Let me refer you to the classic work of John Hilton on "Rest and Pain," the perusal of which cannot fail to teach you much that will assist you in your everyday work, especially in regard to the subject I am now speaking of. You must remember that to be of any avail in such conditions as I have just mentioned the rest must be absolute and continuous, and you must be ever on your guard lest your patients continue to move injured and diseased parts, in spite of the most cunningly devised apparatus you or the surgeon can contrive. Children are especially ingenious in defeating all attempts made to fix their limbs or spinal column.

Extension Apparatus.—It is important that every nurse should know how to prepare this apparatus, as well as how to apply it, as this duty is usually left to her.

Uses.—Extension is applied to the lower limb in almost every condition in which it is necessary to keep that part at rest. Most frequently it is used in strumous disease of the hip-joint, but often also in the same condition of the knee. In such affections also as abscess of the thigh, psoas abscess, and so on, this means of keeping the limb at rest is found very efficient. Many surgeons nowadays use extension, with or without splints, in the treatment of fracture of the femur. It is especially useful in children, in whom the long splint, which was the older way of fixing the part, is so apt to be wet and soiled. The necessary cleaning of the child also interfered very much with the apparatus, and so prevented the maintenance of the absolute rest necessary in the treatment of such cases.

Materials Required.—(1) A quantity of strong mole-skin adhesive plaster; (2) a quantity of strong broad tape; (3) a quantity of boracic lint bandage; (4) an ordinary domette bandage; (5) scissors; (6) strong needle and thread, and tape measure; (7) safety pins; (8) a square piece of wood with a hole in the middle, and leather strap with buckle fixed to each side; (9) apparatus to fix on foot of bed; (10) weights attached to rope; (11) blocks to raise foot of bed; (12) cage; (13) means of heating plaster.

### Method of Preparing the Materials.

The Plaster.—Perhaps the best way to begin the preparation of the plaster is by making a paper shape of it first, and then cutting the plaster from this pattern. Measure from the middle of the thigh to the sole of the foot. This is the length of the plaster. The breadth is about half the circumference of the limb, which is, of course, greater above than below. You must, therefore, shape your plaster accordingly. At the level of the upper end of the malleolus or ankle-bone the plaster should be cut (or folded) so as just to equal the width of the tape used. At the upper end divide the plaster longitudinally into three tails, each about $2\frac{1}{2}$ to 3 inches long. The reason for this you will see presently. Then all along the edge of the plaster make a series of short nicks with the scissors. These permit of closer apposition of the plaster with the leg. Two such pieces of plaster are necessary, one for each side of the limb.

The Tape.—This should be strong twilled tape, about one inch wide and one and a-half foot long. Sew the tape firmly to the narrow lower end of the plaster, attaching the two for about an inch of their length.

The Boracic Bandage should be long enough to cover in the whole limb from the toes up to the middle of the thigh, and of the appropriate breadth for the limb to which it is to be applied.

*By kind permission of Messrs. Weiss and Sons.*

Method of Applying the Extension Apparatus.

The plaster may be applied next the skin of the limb, but as this is rather uncomfortable for the patient, and as it causes considerable pain by pulling on the hairs when being removed, it is better to apply it outside of the boracic lint bandage. I shall, however, describe both methods.

1. Next the Skin.—The limb having been thoroughly washed with soap and water, the hairs should be shaved off, to avoid unnecessary pain when the plaster comes to be removed. Apply a few turns of domette bandage round the foot and ankle, to prevent pressure of the tapes on the malleoli. Next heat the plaster by applying its non-adhesive surface to the side of a vessel containing hot water, or by other means. In warm weather the heat of the body is quite sufficient. Apply the plaster to the surface of the skin so that it adheres evenly, from the upper edge of the patella or knee-pan to the upper level of the malleoli. By folding the nicks you have made along the edge, you will get very accurate apposition. The three tails at the upper end are not to stick to the skin. Now apply your domette bandage from the toes upwards as far as the plaster goes, leaving out the tapes at the lower end. When you reach the three tails at the upper end, fold down the middle one so that it lies on the bandage, bring round another roll, covering in this tail, then fold down the remaining two, and cover them with another turn of bandage. By this means the plaster is not only adherent to the limb, but also to the bandage, thus increasing its security. Lastly, fix the bandage by a safety pin inserted in the long axis of the bandage.

2. ON THE TOP OF A BANDAGE.—The advantages of this method are that the apparatus is more comfortable for the patient, there is no pain in removing it, and the limb is left clean and comfortable. It is advisable to use boracic lint bandages next the skin, because they are non-irritating, and at the same time are toxic to fleas, &c., which is often important. It is essential that the bandage be carefully and accurately applied, else the plaster is apt to drag it down when the weights are attached. The plaster is applied just as in the last case, leaving the tails free above, and avoiding the malleoli below; and covered over by a domette bandage with the same precautions, and dealing with the "tails" in the same way as before. The plaster is allowed to get fixed for a few hours before adding the weights.

APPARATUS TO FIX ON FOOT OF BED.—Perhaps the most convenient form of apparatus for this purpose consists of a framework which is hooked on to the foot of the bed. Through the flat board which forms the top of this a short piece of wood is passed. This is surmounted by a pulley, and can be raised or lowered to suit the height of the bed to which it is applied. The rope is passed through this pulley, and then through the hole in the centre of the small square piece of wood to which the leather straps are attached. The tape on the plaster is then fixed to these straps, and the appropriate weights attached to the rope. (Vide diagram.) A simpler apparatus may easily be extemporised by lashing two strong uprights to the foot of the bed or crib, and using an empty cotton reel suspended between them on a stout piece of wire or wood as a pulley.

THE WEIGHTS.—These may be either ordinary weights used in commerce, or masses of lead or iron, each weighing a half to one pound, and perforated in the middle for attaching them to the rope. Bags of sand or leaden shot of known weight may be used when others are not available. The plaster should always be allowed to fix for an hour or two before the weights are attached, otherwise it is very apt to be dragged off. A light weight should be put on at first, and gradually increased, as by this means the muscles are not unduly fatigued at once. For a child of six years begin with 2 lb., and go up to about 3 lb. or 4 lb.; for an adult, begin with 4 lb. or 5 lb., and go up to 8 lb. or 10 lb.

RAISE THE FOOT OF THE BED.—The object of this is to use the weight of the patient's body as a "counter-extension," otherwise the weights attached to the limbs would tend to pull the patient to the foot of the bed rather than to pull on the limb, and so retain the diseased joint at rest. In hospital suitable blocks are always available for this purpose, but in private one or two bricks or blocks of wood will serve equally well.

CAGE.—After all is fixed, a cage should be put on *over the rope and tapes* to protect them from the pressure of the bed clothes, which, of course, would prevent the proper traction of the weights.

AFTER-TREATMENT.—As a rule, the comfort resulting to the patient from the apparatus makes him lie quiet, as only when he does so is he free from pain. In the early stages of joint disease, however, when the patient has not much pain, he may be restless and attempt to move about. A long splint, applied from the axilla to beyond the foot on the *sound* side, will usually keep a child at rest; or, if necessary, he may be tied down by means of a sheet passed over his chest and secured to the sides of the bed. As in all cases in which the leg is bandaged up, the toes should be left exposed, and should be examined periodically to make sure that no part of the apparatus is interfering with the circulation of the limb. Should the toes get cold or blue, or show any other evidence of obstruction to the circulation, the cause must be sought for, found, and rectified at once.

### The Eastern Hospital Scandal.

LAST week the evidence of Dr. Collie was heard. Asked by Mr. Gye about the food, Dr. Collie replied: I see the food nearly every morning in the kitchen before it is cooked. I see a sample of the milk, and usually taste it. I see the bread generally as it comes in. I usually see the fish in the kitchen. —Do you remember Simpkin being in the hospital? I do. I remember his calling my attention to the fish he had. He said the fish stank, and was not fit for human food. I examined the fish. It was a piece of fresh haddock, very nicely cooked, as I thought. I tasted it. It did not stink to my sense of smell. No other patients complained to me on that occasion about their fish. I went into the kitchen, and told the cook to send him up two lamb chops and some potato. —Is there any truth in the statement that any of the patients were deprived of their food because it was bad or insufficient? Never, but I have heard some complaints as to the meat being too fat. The surgical cases were dealt with by the medical officers of the ward. Cross-examined by Mr. Eldridge: The work of the nursing staff generally was very hard, and sometimes he had a difficulty in getting his requirements granted by the Committee. He was not aware that he had been conducting the hospital on rather economical lines. It was a very wide question whether 7d. per head per day was sufficient, but he had nothing to do with the cost. It might be 3d. per head for all he knew or cared. The patients got the things he ordered for them, and there his duty ended. Mr. Gye: Do the nurses ever have to deal with a delirious adult patient? Yes, but if it is a bad case we put a male attendant to look after him. My opinion of Nurse Halkin is that she is a very good ward nurse, and kept her ward very tidy. She was not sympathetic with her patients, and I do not think a nurse can be a first-class nurse if she is not sympathetic. Mr. Gye: Do you remember her calling your attention to certain beds in St. George's day-room which she said had not been disinfected? Yes; but I found they had been disinfected. I never heard anything about scarlet fever breaking out in those beds until I read Nurse Halkin's letter. As a matter of fact, scarlet fever does break out in diphtheria wards. I do not go round the wards in the clothes I wear out of the hospital. I wear my old clothes in the hospital, and they are too ragged for outside use. Nurse Halkin was continually quarrelling with her assistant nurses, or they with her. I spoke at last to her about it, and told her it could not go on any longer—that I was tired of these complaints. After that there were no more complaints. When she left I gave no orders that she should be hissed out of the hospital. We have no nurses who cannot read or write, but the same remark does not apply to all the assistant nurses, for we have one assistant nurse who cannot write. We have very few assistant nurses under eighteen years of age. I have never had Night Superintendent Dowsett reported to me for being drunk, and it has never been reported to me that she was picked up unconscious.—You say you do not believe in disinfectants? No; the only disinfectants I believe in are soap and water and fresh air. I also attach no importance to wrappers, and only wear them to satisfy the public mind, on the same principle that you give a hysterical patient pills and make her sleep.

The inquiry has now concluded.

### The Sarah Acland Home for Nurses, Oxford.

We are of opinion that the place which nursing institutions all over the country may properly fill has not yet been clearly defined by their managers or the public. There seems reason to hope that a nursing institution, by the adoption of a modified form of the pay system, may gradually become the means of enforcing much-needed reformations in the existing scheme of medical relief all over the country. The committee of a nursing institution can make a wise selection of a good site, for all are open to their choice. They can gradually provide more and more pay beds for the accommodation of patients as circumstances or the demand may necessitate, and thus do a real service to a great number of people when sick, and to the members of the medical profession by taking in patients at a remunerative but moderate rate of payment, and allowing them to be attended by their own doctors. It is quite clear that the medical treatment should be provided outside the nursing institution altogether, and that the patients and their medical attendants should be left free to arrange such terms as may be found mutually satisfactory. In a wise elaboration of this combination of nursing and the treatment of paying patients would be found one practical solution to many of the existing evils attending hospital treatment in the present day. We propose to return to the subject on another occasion, but the Acland Home is so wisely conducted as to enforce the principles here formulated, and we could not, therefore, pass them by without notice.

### Origin and General Aspects.

The Sarah Acland Home at Oxford was started as a memorial to the wife of Sir Henry Acland, the eminent physician, whose services to the profession and to nurses are so well-known and widely appreciated. It is situated in Wellington Square, where the freehold of two houses has been acquired by the Committee. The organisation includes District Nursing, a Maternity Branch, Private Nursing, and the conduct of Medical and Surgical Homes, where 80 patients were treated during the last year. The Homes are plainly furnished, and each district nurse has a room to herself. There is a common sitting-room, a small library, and every possible provision seems to be made for the comfort and well-being of the nurses. We only saw some of the district nurses, who seem to get through a large amount of work in a quiet and effective way, to the no small advantage of the sick poor of Oxford. The excellence of the district nurses at the present time is in no small degree due to the devoted and effectual aid rendered by Mrs. T. H. Green as Assistant District Superintendent during the last four years. Four hundred and thirty-eight cases have been nursed during the past year, and 12,399 visits have been paid to the sick. In sixty-nine cases the nurses were supplied for night duty. It may be useful to mention an act of courtesy and liberality on the part of the manager of the Oxford Tramways Company, who has given a free pass to the Lady Superintendent, and special passes at reduced rates to the five district nurses when engaged in their work. No branch of the Sarah Acland Home merits greater commendation than the "skilful and loving tendance of the sick poor" which "constitutes in the chiefest sense the tribute to her in whose memory the Home was founded." Forty-six cases were attended in the maternity branch. The Committee express their indebtedness to those medical men who kindly extended their aid to the midwife when required. The private nursing staff consists of twenty-five members, and it has attended one hundred and eighty-five cases during the year. The charges vary from one guinea for ordinary cases to two guineas for infectious, massage, or mental cases. Five shillings a night is charged for night duty, and a disinfecting fee is now imposed.

### The Private Hospital.

The rooms devoted to the accommodation of paying patients have commended themselves to the medical profession and the public in Oxford. The demand, indeed, during the autumn and winter was so great that patients had to be refused admission, and applications were received from a distance as well as from the University and city. Indeed, the Committee are making an effort to secure ground on which to erect a building specially planned for medical and surgical cases, a large number of which now seek admittance to the private hospital. As we have already said, there can be no doubt that very many persons who are ineligible from their circumstances and position for admission to the Radcliffe Infirmary, and whose homes, or college rooms, do not admit of their being efficiently nursed at home, may be usefully and profitably provided for in a paying ward, such as that provided at the Acland Home. We inspected these wards, and found them airy and comfortable. The rules are modelled upon those of the first Home Hospital (Fitzroy House, Fitzroy Square), and the terms vary from three guineas per week for a separate room, to £1 10s. per week in a room with two beds. There are one or two large front rooms, for which a charge of four guineas is made. These terms include board and ordinary nursing, and everything except stimulants, mineral waters, medicines, and surgical dressings. Patients requiring the undivided attention of one or more nurses must make special arrangements. All patients in the Home are under the charge of their own medical officers. Each medical attendant is considered responsible for the professional care of his patient, and is to provide for such professional assistance as may be required during his absence. The Committee do not hold themselves responsible for the fees of the medical attendant, as that is a matter altogether outside their jurisdiction.

### The Matron and Nurses.

No one can visit the Acland Home without being struck by the earnest purpose and intelligent interest which characterises every aspect of the administration under Miss Denniston's management. Familiar with every detail, impressed with the usefulness of the institution and its powers of extension, knowing her nurses well, and taking a keen interest in their welfare as well as in that of the institution, it is a pleasure to meet the Matron and to realise, as one cannot fail to do, the purpose and fulness of her knowledge. We have already said something of the district nurses in passing, and from all we learned and saw we can well believe they are widely popular in Oxford amongst the poor, whom they do so much to help in the time of need. We were sorry to hear that the Committee had decided not to formulate any definite system of pensions, but to give each nurse a sum per annum to do what she liked with. We venture to think that this plan is neither desirable nor fair. Not desirable, because it fails to bring to bear, in the interests of the nurses, the experience and knowledge of the Committee so as to secure an adequate provision for each nurse against sickness or old age. Not fair, because nurses are often drawn from a class which makes the members of their families regard them as in a position of relative prosperity, and so causes the friends to deplete a nurse's earnings by constant drains upon her small resources. Hence it is quite possible that, in spite of an institution giving each nurse on its staff a sum, say, of £4 a-year, for the purposes of a pension if it be left absolutely at the nurse's own disposal, it will be found that in the day of sickness and old age not 5 per cent. of the nurses belonging to such an institution will have made any adequate provision, or have funds in hand to pay for their maintenance. The managers of the Acland Home take so keen an interest in its welfare and its staff, that we hope it is not too late for them to reconsider this proposal which we are confident will be found in practice to fail in its purpose, and so to prove unsatisfactory to the institution as well as to the nurses employed.

The Work and its Future.

Of the work of the Acland Home, it may be said that few institutions accomplish so much for so small an outlay as £2,000 a-year. Of the whole income of £2,100, nearly £1,500, or about 75 per cent., is derived from patients' payments and nurses' earnings. The interest taken in the Home by the Committee, which consists, by the way, entirely of ladies, is remarkable, and is another proof of the devotion of women to public work when they undertake it and have faith in it. There can be no question judging from the success which has already attended the establishment of a relatively small number of paying wards, that when the new medical and surgical home has been built it will prove widely popular, and gradually become one of the recognised and most valued institutions in the City of Oxford. We are not quite clear if there are sufficient district nurses to thoroughly undertake all the work which the needs of the poor require in the City of Oxford, but so far as that work has been overtaken, it is done excellently well. This circumstance should lead to an extension of the district work to the fullest extent necessary to provide that no one amongst the sick poor of Oxford shall henceforth be left without the skilled and loving attendance of a trained and efficient nurse. If this be accomplis ed, the memory of Sarah Acland will be for ever perpetuated to the no small advantage of the poor whom she loved so well, and to the eternal credit of all who have had anything to do with the establishment and administration of this excellent institution.

### Everybody's Opinion.

*[Correspondence on all subjects is invited, but we cannot in any way be responsible for the opinions expressed by our correspondents. No communications can be entertained if the name and address of the correspondent is not given, or unless one side of the paper only be written on.]*

### FLOWERS FOR HOSPITALS.

"A Nurse" writes: Will any nurses who may be working in country places, where wild flowers do grow, remember the poor London hospitals? Cowslips are just coming out, and all they want is to be tied in bunches with a bit of worsted, rolled up tight in brown paper, and posted at once.

### A PRO.'S GRIEVANCES.

"Poor Pro." writes: Would you kindly give me a small corner in your valuable paper for a fact about a probationer's year in one of cur largest training schools for nurses in the North of England? A probationer pays £20 for her training, and provides her own uniform, &c. In return, she is supposed to be thoroughly trained in medical and surgical work, and besides having a month in maternity wards, she is suppoted to have a little of children's nursing. But if parents and guardians knew the real training some of these poor girls receive, they would look further into the working system of the place, before throwing away their money, and sending young girls to have their health ruined, as it unfortunately is in many cases, then sent back at the year's end as fully qualified nurses, when, in reality, they know little more than they did the first day they entered the building. To give you a case in point: Nurse S—, we will call her, sent by one of the many nursing institutions in the South of England to be trained for them, was put in the men's crib wards, where she was left over two months. Then she had five months in the mothers' and babies' ward, where it was really nursery-maid's work, as the babies were from three days to eighteen months' old. Many of them were ill 'tis true, and many a poor baby might have been saved a deal of unnecessary suffering, had there been a competent nurse in charge of the wards. For the head nurse had no idea how to treat children. Could she have, when she had never been in children's wards until she was sent to take charge of them? And how could she teach her probationer what she did not know herself? Then came two months' infirm, which is nothing more or less than keeping old people clean. Now begins the training which should have commenced six months' ago. She is sent to medical wards, where there is really something to learn, and she eager to learn it; there she was left three weeks. Afterwards comes surgical, of which she gets six weeks. Lastly came her month in maternity wards, for which the home stipulates; even that was incomplete, as she only saw the cases, and was sent to general nursing between. Her twelve months are over. She is sent back to the home a fully qualified nurse! Now she goes into private families, and valuable lives are entrusted to her care. Are the heads of that institution wronging these girls, the homes they belong to, or the public?

### The Registration of Nurses.

The following correspondence has passed between the Honorary Secretary of the Royal British Nursing Association and the Treasurer of St. Thomas's Hospital, as the representative of the Nurse Training Schools :—

From the Honorary Secretary of the R.B.N.A. to the Treasurer of St. Thomas's Hospital.

As a meeting of the General Council of the Royal British Nurses' Association has been convened by Her Royal Highness the President to consider the opposition of yourself and others to the incorporation of the Association under the 23rd section of the Companies' Act of 1867, I beg to inform you that it will be held at 20, Hanover Square, London, on Thursday, April 16th, at 5 p.m., and that you or any other representatives of Nurse Training Schools who desire to be present at this meeting will be welcomed.

(Signed) Bedford Fenwick, Hon. Secretary.
April 13th, 1891.

From the Treasurer of St. Thomas's Hospital to the Honorary Secretary of the R.B.N.A.

I have to acknowledge receipt of your invitation to join a discussion on the question of the opposition by all the leading Nurse Training Schools of the country to the application of the Royal British Nurses' Association for a Board of Trade licence.

No one can regret more sincerely than I do that there should be the necessity for such an opposition. The Royal British Nurses' Association is alone to blame for this most unfortunate state of disagreement, as its actions have been in direct opposition to the expressed opinion of nearly all those who knew most of the subject.

Personally I feel it is much to be regretted that our Most Gracious Sovereign's name should have been allowed to be associated with a movement which those most truly and historically interested in this nationally important work believe to be inimical to its welfare, and at the same time misleading rather than otherwise to the public.

I may add that the objections to your proceedings are fully stated in the memorials to the President of the Board of Trade, and our future action will be guided by the Committee of Representatives of the Nurse Training Schools now in communication with the Board of Trade. I feel, therefore, that my presence at your meeting would serve no useful purpose.

(Signed) J. G. Wainwright, Treasurer.
St. Thomas's Hospital, April 16th, 1891.

### Presentations.

Mr. C. F. Althorp, on resigning the post of House Surgeon to Bradford Infirmary, was the recipient of several presentations. Mr. Briggs, of the Board of Management, made a speech, acknowledging the feeling of regard all had for Mr. Althorp. The presentation was then made by the Lady Superintendent of a walnut writing-cabinet from the nursing staff, a table gong from the servants, and a china afternoon tea-service from the patients in the Jenny Philipp and Macturk Wards. Mr. Althorp, in appropriate terms, acknowledged the gifts, which, he said, would often remind him of the happy days spent in the service of the institution. Mr. John Bennett afterwards gave before the nurses a magic lantern entertainment descriptive of a tour through Egypt and up the Nile, each picture as it was put on the screen being described by Mr. Briggs. Mr. Maw, Secretary, Dr. Firth, House Physician, and others were present.

On the 11th inst. Mrs. Crossley, Night Superintendent of St. George's-in-the-East Infirmary, was presented by the nurses with a handsome marble clock on her resignation.

On April 16th Surgeon Ormsby was presented by the Dublin Red Cross Sisters with an address and handsome claret jug, in recognition of his services in establishing the order. There was a large gathering of medical men, and Viscount Powerscourt took the chair. Surgeon Ormsby, in replying, complimented the Sisters on their work, and especially gave his thanks to Sister Lyons, the head of the order.

### Pat.

I.

"Christmas Day, then, darlint?"

"All right, Pat; Christmas Day, then, we'll be spliced." The speakers were an Irishman and his sweetheart. The scene was a small room in Whitechapel. Pat was as bonny a specimen of an Irishman as anyone might wish to see, with curly brown hair, bright, mischievous blue eyes, and a tall, erect figure. His sweetheart, Milly, was not worthy of admiration to any eyes but Pat's. Just a type of an East-end girl, with the big, dirty fringe so much affected by people of that class, bold brown eyes, short and squat, very much the reverse in every way of Pat; perhaps therein lay the attraction. So Christmas Day was fixed for the wedding, and certainly Pat was in love, and Milly thought she was.

Pat, light-hearted and gay, thinking more of his sweetheart than where he was going, chose an unlucky moment to cross a street. A din in his ears, one second of acute agony, and Pat was taken up unconscious; a tram had gone over his leg. A slight bustle in the receiving-room of the great hospital of the East-end as two policemen bore in the unconcious Pat. "Here comes a regular Saturday night smash-up," remarked the receiving room officer as he turned to examine the case. The house surgeon on duty then came forward, having been sent for by the receiving-room officer. Pat was soon despatched on a stretcher to the accident ward "Worcester," the porter carrying in his hand a paper, which he delivered up to the nurse in charge, who read "Compound fracture of tibia and fibula." A screen was put round the bed, and Pat still only semi-conscious, was soon washed and made realy for the visit of the house surgeon.

"Amputation; the fellow must submit; explain to him, Sister, it is loss of limb or life "—and the Sister explained to him. Things you see had not gone well with Pat, and gladly as the head surgeon would have saved the limb, he could not, it was beyond his skill. But Pat, poor Pat, would not submit, "his darlint, his Milly shure and she niver would marry wi' a one-legged mon."

And Milly—the first time she came to see him, she gave way to the most noisy violent grief; next visiting day she was quieter, and agreed with Pat the wedding must be deferred; the third visiting day, Pat had asked her if his leg had to come off "Would she marry wi' a one legged mon," and Milly's answer, shame to her, had been "No."

"Izard Ward," the ward set apart for erysipelas, in the male ward lies an Irishman, with curly brown hair and sad wistful blue eyes. Alas! for poor Pat, erysipelas has set up in the wounded limb. But above, beyond, far worse than the pain of his limb, is the ache the thought of Milly brings. Pat has now consented to the loss of his leg, and drearily as he lies on his bed does he picture his future, the future of "a mon wi' one leg."

When the erysipelas has run its course, Pat is to be thoroughly disinfected, and to go back to his old ward, "Worcester," and have the amputation performed from there.

That was Pat's wish, to go back to his own ward. He wished to be with his "Sister," the one who had smiled and spoken kindly to him when he first came in; small wonder he wished that, for Sister Worcester was one of the prettiest, best little women in the hospital.

Mr. Grey's house was in Wiltshire, he was the Squire of the small parish of Shirley. His house was known in the parish as "Squire's 'Ouse" or "the 'Ouse." It boasted of two entrances, each guarded by a lodge. The occupant of one of these lodges was "a mon wi' one leg." So here we meet again our friend Pat in more prosperous circumstances; the post had been got for him, by his dresser in the hospital, who had been interested in Pat, and being a nephew of Mr. Grey's, he had recommended, nay more, begged him to give Pat a trial, which he had.

We must pass over three years, and then again review Pat. Pat no longer a lonely bachelor but a husband and father; he had wedded a housemaid from "the 'Ouse," a bright respectable girl. Pat's baby is a little girl and has been baptized "Milly," for he tells his wife he "kind o' loikes" the name. If I might be allowed to point a moral to such a short tale, it would be this: That every patient is an individual, not only a number, 52 for instance, or a case "Tib. and Fib.," but has its own life history, troubles, and disappointments, and the same need and demand for sympathy as any one of us.

### Appointment.

Miss Noemie Armit, Matron of the Bolton Infirmary and Dispensary, has been almost unanimously elected Matron to St. Saviour's Infirmary, East Dulwich, London.

### Notes and Queries.

### Queries.

(5) *An Epileptic Case.*—Miss Bott, Hanbury Hall, Burton-on-Trent, will feel grateful to anyone who can inform her of an institution that will receive a little girl, six years of age, suffering from epileptic fits, who is blind and cannot stand or speak in consequence. The parents are respectable working people, in straitened circumstances through ill-health.

(6) *Nursing Ethics.*—Is it allowable in strict honour for a Matron or Sister-in-Charge to apply for a more lucrative or advantageous post before resigning her present work, or before hinting to anyone connected with it that she means to look out for something better?—*Sister X.*

(7) *Home Wanted.*—For a lame old man, aged 70; could pay 10s. a-week. Miss N. C. Pike, Sandrock Hotel, Niton, Isle of Wight.

### Answers.

*Edwards.*—We never prescribe.

(2) *District Nursing.*—In the town of Loughborough the poor people pay the district nursing society according to their means for the services of the nurse. Some pay as low as 6d. a-week. Nurse Clarke, of Pembroke, writes to say her patients make small payments.

*L. C. H.*—The "Annual" can be had by sending a postal order for 3s. to this office (140, Strand, W.C.).

*A Constant Reader.*—Apply to the Matron of Queen Charlotte's Hospital. You will have to pay 10 guineas for eight weeks' training, or 25 guineas for three months' training.

*Gertrude.*—"Hoblyn's Dictionary of Medical Terms," Whittaker and Co., Paternoster Square, E.C. Price 10s. 6d.

### Amusements and Relaxation.

SPECIAL NOTICE TO CORRESPONDENTS.

Second Quarterly Word Competition commenced April 4th, ends June 27th, 1891.

Competitors can enter for *all* quarterly competitions, but no competitor can take more than *one first prize* or *two* prizes of any kind during the year.

The words for dissection for this, the FOURTH week of the quarter, being "PORTUGAL."

| Names. | April 16th. | Totals. | Names | April 16th. | Totals. |
|---|---|---|---|---|---|
| Christie | 75 | 96 | M. G. | 66 | 83 |
| Patience | 76 | 97 | Ivanhoe | 59 | 75 |
| Agamemnon | 77 | 98 | Weta | 58 | 74 |
| Hope | 77 | 97 | Lady Betty | 77 | 93 |
| Reldas | 76 | 96 | Mortal | 53 | 68 |
| Lightowlers | 75 | 95 | Little Eliza | 62 | 77 |
| Nurse J. S. | 48 | 67 | Dora | 58 | 73 |
| Qu'appelle | 74 | 93 | Ladybird | 6[illegible] | 74 |
| Jenny Wren | 66 | 84 | Psyche | 58 | 71 |
| Wyameris | 77 | 95 | Ugug | 59 | 72 |
| Pa gnton | 59 | 76 | Harrie | — | 10 |
| Theta | 75 | 92 | Grannie | 59 | 68 |
| Success | — | 17 | Eale | 54 | 63 |
| Tired | 57 | 74 | Grimalkin | 45 | 53 |

For Rules see The Hospital April 4th, 1891.

## Figures and Tables

**Figure f1:**